# GABAergic cell transplants in the anterior cingulate cortex reduce neuropathic pain aversiveness

**DOI:** 10.1093/brain/awz203

**Published:** 2019-07-18

**Authors:** Dina L Juarez-Salinas, Joao M Braz, Alexander Etlin, Steven Gee, Vikaas Sohal, Allan I Basbaum

**Affiliations:** 1 Department Anatomy, University California San Francisco, San Francisco, CA, USA; 2 Department Psychiatry, University California San Francisco, San Francisco, CA, USA

**Keywords:** conditioned place preference, chemotherapy-induced neuropathic pain, pain aversiveness, medial ganglionic eminence GABAergic cell transplants, gabapentin

## Abstract

Dysfunction of inhibitory circuits in the rostral anterior cingulate cortex underlies the affective (aversive), but not the sensory-discriminative features (hypersensitivity) of the pain experience. To restore inhibitory controls, we transplanted inhibitory interneuron progenitor cells into the rostral anterior cingulate cortex in a chemotherapy-induced neuropathic pain model. The transplants integrated, exerted a GABA-A mediated inhibition of host pyramidal cells and blocked gabapentin preference (i.e. relieved ongoing pain) in a conditioned place preference paradigm. Surprisingly, pain aversiveness persisted when the transplants populated both the rostral and posterior anterior cingulate cortex. We conclude that selective and long lasting inhibition of the rostral anterior cingulate cortex, in the mouse, has a profound pain relieving effect against nerve injury-induced neuropathic pain. However, the interplay between the rostral and posterior anterior cingulate cortices must be considered when examining circuits that influence ongoing pain and pain aversiveness.

## Teaser

Peripheral nerve injury-induced neuropathic pain has both sensory discriminative and affective features. Juarez-Salinas *et al.* report that transplanting embryonic GABAergic progenitor cells from the medial ganglionic eminence into the rostral anterior cingulate cortex reduces ongoing pain aversiveness in a mouse model of chemotherapy (paclitaxel)-induced neuropathic pain.

## Introduction

The perception of pain has two major features: a sensory-discriminative component that processes the modality, location and intensity of the noxious stimulus, and an affective/emotional component that is critical to the aversive/unpleasant quality of an ongoing pain experience. Circuits in the primary somatosensory cortex (S1) mediate the sensory-discriminative component (Kanda *et al.*, 2000). In contrast, the rostral anterior cingulate cortex (rACC), insular cortex and amygdala are implicated in processing of the affective component of the pain experience (Price, 2000). Strikingly, patients who have damage to the ACC or cingulum bundle, a major afferent pathway interconnecting the frontal cortex with the ACC, typically find painful stimuli no longer bothersome (Foltz and White, 1962). Likewise, surgical destruction of the rACC or cingulum bundle provides significant relief in patients with ongoing pain (Foltz and White, 1962; Ballantine *et al.*, 1967; Sharma, 1973; Hurt and Ballantine, 1974). Interestingly, deep brain stimulation of the ACC has also proven effective against the affective component in patients with a variety of neuropathic pain conditions (Boccard *et al.*, 2017). One view of the contribution of pain-related activity in the ACC is that the aversiveness that results from ACC activity serves as a teaching signal (punishment) that shapes subsequent behaviour, for example, avoidance or escape from the precipitating stimulus. However, when aversiveness persists, even when the injury has resolved, pain loses this value, becomes chronic, and is now maladaptive.

As lesions of the ACC reduce the aversiveness that occurs in various rodent neuropathic pain models, it has been proposed that injury-induced hyperactivity, secondary to loss of GABAergic inhibitory controls, underlies the contribution of the ACC to pain aversiveness. For example, peripheral nerve injury induces ACC astrocyte upregulation of GABA transporters (GATs), which remove GABA from the synaptic cleft (Narita *et al.*, 2011), decreases GABA release (Narita *et al.*, 2011) and reduces inhibitory postsynaptic currents in ACC pyramidal neurons (Blom *et al.*, 2014). Consistent with a deficit in GABA signalling in the ACC, other studies reported electrophysiological and molecular changes, including increased expression of the GABA transporter 1 in a chemotherapy-induced neuropathic pain model (Masocha, 2015; Nashawi *et al.*, 2016). Other studies reported that injection of GABA receptor agonists into the rACC of nerve-injured mice reduces pain aversiveness following nerve injury. The latter was demonstrated both by a reduction of noxious stimulus-provoked conditioned place avoidance (CPA) (LaGraize and Fuchs, 2007) and of fear conditioning in response to painful foot-shock (Tang *et al.*, 2005).

Taken together these studies suggest that a prolonged increase of GABA-mediated signalling in the rACC should alleviate a tonic state of aversiveness associated with neuropathic pain. Unfortunately, traditional pharmacological approaches to reinstating inhibitory controls are neither long term, nor exerted within defined circuits of the ACC. To address these limitations, here we adopted a cell transplantation approach that enhances local GABAergic inhibition (Braz *et al.*, 2012; Southwell *et al.*, 2014). In our earlier studies, we transplanted GABAergic progenitor cells derived from the medial ganglionic eminence (MGE) into the dorsal horn of mice in which there was a profound mechanical hypersensitivity produced in different models of neuropathic pain (Braz *et al.*, 2015*b*). The transplants not only integrated synaptically into host circuits of adult mice (Etlin *et al.*, 2016; Llewellyn-Smith *et al.*, 2018) in a random fashion, but also completely reversed the mechanical hypersensitivity. In the present study, we asked whether transplants into the rACC can reverse the affective component of the neuropathic pain phenotype and whether this can occur without concurrently blocking its sensory-discriminative component, namely mechanical hypersensitivity.

To document pain aversiveness, we used an analgesia-induced conditioned place preference (CPP) paradigm (King *et al.*, 2009) in which animals that experience pain associate one side of an apparatus with an analgesic agent, namely gabapentin. Importantly, gabapentin is not inherently rewarding. Thus, only in the setting of ongoing pain do the animals show a preference for the gabapentin, as it is the pain-relieving effect that provides the reward. In the present report, we used a chemotherapy (paclitaxel)-induced neuropathic pain model associated with profound mechanical and thermal hypersensitivity (Smith *et al.*, 2004; Braz *et al.*, 2015*b*). We report that the transplants blocked preference for gabapentin, despite ongoing mechanical hypersensitivity. In other words, by enhancing GABAergic inhibitory tone in the ACC, the transplants provide long-term pain relief, namely a reduction in aversiveness. Unexpectedly, we report that MGE transplants are only effective when they are restricted to the rACC. When there was significant migration of the transplanted cells into the posterior ACC (pACC) the pain relief was lost.

## Materials and methods

### Mouse lines

All experiments were reviewed and approved by the Institutional Care and Animal Use Committee at the University of California San Francisco. MGE cells were dissected from transgenic mice that express GFP under the control of the *GAD67* promoter (Gad1; Tamamaki *et al.*, 2003). All transplants were performed on male mice (6–8 weeks old; C57BL6/J; Jackson Labs: Strain 664). MGE donor mice were of a CD1xC57BL6/J background. For some experiments, MGE cells were dissected from double-transgenic I12b-ChR2 mice, which express the light-sensitive cation channel channelrhodopsin 2 (ChR2) selectively in the forebrain (Potter *et al.*, 2009; Madisen *et al.*, 2012).

### Transplantation of medial ganglionic eminence cells

We prepared and transplanted 50 000 MGE cells into the ACC of 6–8-week-old mice as described previously (Braz *et al.*, 2012). Animals were killed at 4–5 weeks post-transplantation. Importantly, none of the transplanted animals exhibited signs of motor impairment. For some anatomical tracing studies, naive mice (*n = *4) were transplanted with MGE cells that were genetically modified so as to express tdTomato, rabies glycoprotein, and the TVA receptor. In these experiments, EnvA-GFP-rabies virus (a gift of Dr Fan Wang at Duke University) was injected into the transplant region (∼10^6^ total pfu) 30 days after transplant of the MGE cells (Braz *et al.*, 2015*b*).

### Antibodies and immunohistochemistry

We used the following antibodies: rabbit anti-GFP (1:2000; Molecular Probes), chicken anti-GFP (1:2000; Abcam), mouse anti-parvalbumin (1:2000; Sigma-Aldrich), rabbit anti-GFAP (1:20 000; Dako), rabbit anti-Iba1 (1:1000; Wako), rat anti-somatostatin (1:5000; Millipore), mouse anti-GABA (1:4000; Sigma-Aldrich), and mouse anti-NeuN (1:2000; Chemicon).

Immunohistochemistry was performed as described previously (Braz *et al.*, 2012). Sections were viewed with a Nikon Eclipse fluorescence microscope, and images were collected with a Zeiss camera (Axiocam). High-resolution confocal images taken on a Zeiss confocal confirmed that the immunoreactivity was cytoplasmic (0.8 µm optical sections). Brightness and contrast were adjusted using ImageJ (Version 1.49b).

### Quantification and statistical analysis

GraphPad Prism® 6.0 g software was used to analyse all datasets. CPP Score = (time spent on the gabapentin-paired side on the post-conditioning day) − (time spent on the gabapentin-paired side of the box on the pre-conditioning day). CPA Score = (time spent on the formalin-paired side on the post-conditioning day) − (time spent on the formalin-paired side of the box on the pre-conditioning day). CPP and CPA data were analysed using the Student’s paired *t*-test comparing CPP or CPA scores of control and experimental animals. When comparing across different treatment groups of mice (e.g. control versus pACC lesioned versus rACC lesioned or control versus rACC only versus rACC and pACC) we compared the average change in preference (post-conditioning − pre-conditioning) for the gabapentin-paired chamber of all three groups of mice using a non-parametric Kruskal-Wallis test with Dunn’s multiple comparison correction. Mechanical thresholds before spared nerve injury (SNI)/paclitaxel and those after SNI/paclitaxel in both the lesion and no lesion (control) groups were compared using a repeated measures (RM) 2-way ANOVA followed by Tukey’s multiple comparison test. Duration of hindpaw licking after formalin injection and latency to withdraw in the Hargreave’s test, in mice with and without (control) rACC lesions, were compared using a RM 2-way ANOVA, Sidak’s multiple comparison test. Data are presented as the mean ± standard error of the mean (SEM) licking duration within sequential 5-min bins. Changes in gene expression assessed by quantitative PCR data were analysed across three groups (14 days sham, 14 days post-SNI, and 30 days post-SNI) using a one-way ANOVA, Tukey’s multiple comparisons test for each gene. For all analyses, significance was set to *P* < 0.05.

### Cell counts and quantification

To determine the number of transplanted MGE cells we counted cells from digitized images of GFP-immunoreactive neurons. We counted all cell bodies in 35 transverse sections (35-μm thick) that included the ACC, and estimated percentage 100 × (total GFP+ cells) / (number of cells in the initial transplant). These studies were performed in 31 animals. In a separate group of three animals, we assessed the percentage of surviving transplanted cells that expressed a second marker, imaged on a Carl Zeiss LSM 700 microscope. These calculations were made from a series that contained all transverse sections (separated by 210 μm) that spanned the cortical areas containing transplanted cells. At least 100 GFP+ MGE cells were analysed for each marker, in each animal (*n* = 3), 30 days after transplantation. We calculated the percentage of double-labelled neurons (marker+and GFP+) by dividing the number of double-labelled neurons by the number of single GFP-labelled neurons × 100. Values are given as mean ± standard error (SEM).

### Paclitaxel model of chemotherapy-induced pain

This model was produced as described previously (Braz *et al.*, 2015*b*). Briefly, mice received four injections of 1.0 mg/kg paclitaxel (Sigma) dissolved in dimethyl sulphoxide (DMSO), every other day.

### Quantitative PCR

Fourteen and 30 days after SNI, mice were killed (*n = *4). Naive (vehicle-injected) mice (*n = *4) were also used as controls. We removed rACC tissue bilaterally from 350 μM vibratome sections, using a 1.0-mm diameter punch. We used the RNeasy^®^ Minikit from Qiagen to extract mRNA, after which 200 ng of purified mRNA was reverse-transcribed into cDNA using SuperScript™ II (Invitrogen). See Supplementary material for primer sequences. Messenger RNA levels were quantified with a Realplex2 real-time PCR system (Eppendorf) using SYBR^®^ Green PCR Master Mix (Applied Biosystems). Ratios of gene mRNA to *Gapdh* mRNA were compared and analysed by a one-way ANOVA followed by a Tukey’s multiple comparisons *post hoc* test. Asterisks indicate statistically significant differences between groups:*P < 0.05, **P < 0.01.

### Electrophysiology: slice preparation and recordings

For these experiments, recordings were performed as described previously (Gee *et al.*, 2012; Etlin *et al.*, 2016); see Supplementary material for details.

### Surgical procedures

#### Sciatic nerve injury

To produce mechanical hypersensitivity we used the SNI neuropathic pain model, as described previously (Shields *et al.*, 2003). Briefly, the sural and superficial peroneal branches of the sciatic nerve were transected and ligated distal to the popliteal fossa, sparing the tibial branch.

#### Excitotoxic lesions

Six days following the last paclitaxel administration, we microinjected ibotenic acid (5.0 mM, 500 µl/side, 200 nl/min; Santa Cruz Biotechnology) into the rostral anterior cingulate cortex (0.4 mm, 1.30 mm, −1.8 mm) or the pACC (−0.4 mm, 0.75 mm, −1.8 mm). In control (no lesion) animals, we made an incision of the scalp, after which the skin was closed with staples. All animals were given 6 days to recover before the behavioural experiments were conducted.

### Circuit tracing in the ACC

Here we transplanted the ACC of naive mice with MGE cells that expressed Cre recombinase and the avian TVA receptor, which can be targeted by pseudotyped rabies virus. Thirty days after transplant, we microinjected 500 nl of EnvA-Rabies-GFPΔG virus (Braz *et al.*, 2015*a*) using the same coordinates. Two weeks later, tissues were processed for immunohistochemistry.

### Behavioural testing

In all behavioural experiments, the individual performing the behavioural testing was blind to treatment (saline, medium, ablation or transplant). Prior to analysis of the results, the mechanical and thermal withdrawal thresholds were tested with the von Frey and Hargreaves test, respectively, as described previously (Wang *et al.*, 2013). To assess the magnitude of the mechanical allodynia, we tested the animals once, 7 days after surgery. Mice transplanted with MGE cells were tested again, 30 days after transplant or Dulbecco’s modified Eagle medium (DMEM). Mice that received ibotenic acid lesions had their thresholds tested once more 6 days following the surgery, before conducting the CPP experiments.

#### Formalin test

Nocifensive behaviours (licking, guarding and shaking of the injured paw) was scored in 5-min bins, from 0 to 45 min post-injection, as described previously (Shields *et al.*, 2010)

#### Conditioned place paradigm

To assess ongoing pain, we used an analgesia-induced CPP paradigm, as described previously (Juarez-Salinas *et al.*, 2018); see also Supplementary material.

### Data availability

The data that support the findings of this study are available from the corresponding author, upon reasonable request.

## Results

### The rACC mediates the affective, but not the sensory-discriminative, component of the pain experience in the mouse

Previous studies in the rat demonstrated that lesions of or microinjections of gabapentin into the rACC alleviate the aversive quality of the pain experience, without interrupting its sensory discriminative aspects; i.e. mechanical hypersensitivity persists (Johansen *et al.*, 2001; Qu *et al.*, 2011; Bannister *et al.*, 2017). To determine if this relationship holds true in mice, we first studied the effect of ibotenic acid-induced excitotoxic lesions of the rACC on three measures of the sensory/discriminative component of pain: (i) hindpaw withdrawal latency to a noxious radiant heat stimulus; (ii) nocifensive behaviours induced by hindpaw injection of formalin, a noxious inflammatory stimulus; and (iii) mechanical withdrawal thresholds before and after partial nerve injury. Finally, to examine the effect of rACC lesions on the affective component of pain, we measured formalin-induced avoidance behaviour in a conditioned place aversion setting.

**Figure 1:**
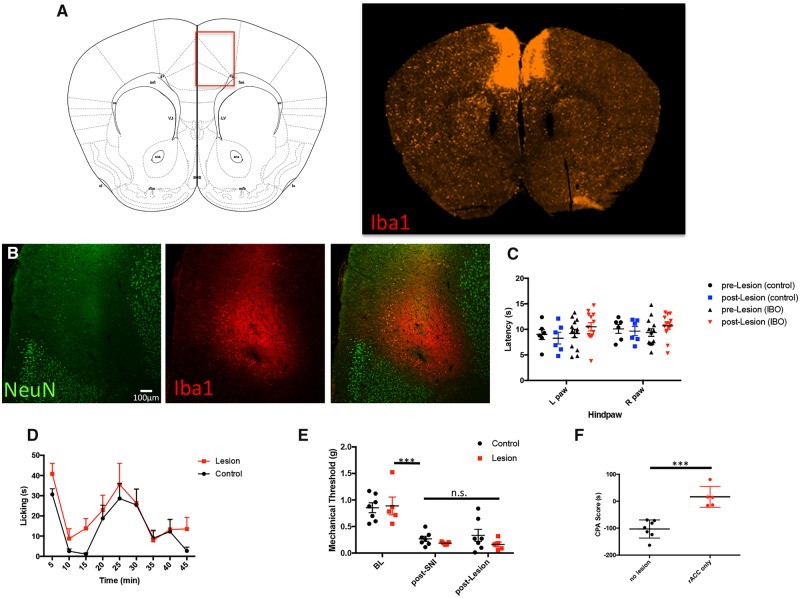
**Rostral ACC lesions do not alter the sensory-discriminative features of the pain experience.** (**A**) Schematic illustrating the location of the rACC (*left*) and Iba1-immunostained section (*right*) illustrating microglial activation induced by the ACC lesion. (**B**) Representative example of excitotoxic lesions of the rACC. Immunostaining with antibodies to NeuN (green) and Iba1 (red) define regions of neuronal loss and activation of microglia in the ACC. (**C**) Left and right hindpaw withdrawal latencies to radiant heat in mice with (*n = *13) and without (*n = *6) rACC ibotenic acid (IBO)-induced lesions. There are no significant differences in acute thermal thresholds between the two groups (RM 2-way ANOVA, Sidak’s multiple comparison test, not significant). (**D**) Duration of hindpaw licking after formalin injection in mice with (red, *n = *12) and without/control (black, *n = *9) rACC lesions. There is no significant difference between the groups (RM 2-way ANOVA, Sidak’s multiple comparison test). Data are presented as the mean ± SEM licking duration within sequential 5-min bins. (**E**) Mechanical threshold ipsilateral to the partial sciatic nerve injury (SNI) before injury (baseline, BL), after injury (post-SNI) and 1 week after rACC lesion (post-Lesion). Mechanical allodynia ipsilateral to the injury did not differ in mice with (*n = *9, red) or without (*n = *7, black) ACC lesions (RM 2-way ANOVA followed by Tukey’s multiple comparison test, ***P < 0.001). Data are represented as mean ± SEM (**F**) Compared to control mice (*n = *7, black), rACC lesioned mice (*n = *5, red) do not show formalin-induced aversion in the CPA test (Student’s *t*-test, ***P < 0.001).

We measured baseline thermal thresholds prior to and 6 days after the rACC lesion (documented by increased microglial Iba1 immunoreactivity and decreased NeuN immunoreactivity; Fig. 1A and B; *n = *13); control animals underwent sham surgery (control, *n = *6), in which only the scalp was exposed. As predicted, we found no difference in baseline thermal thresholds in the control and lesion groups (Fig. 1C; RM 2-way ANOVA, Sidak’s multiple comparison test, not significant). In the formalin test, we also found no difference between the lesion (*n = *12) and control (*n = *9) groups in nocifensive behaviours (paw flinching and licking), at any of the time points (Fig. 1D; RM 2-way ANOVA, Sidak’s multiple comparisons test, not significant). These results are consistent with previous studies in the rat (Johansen *et al.*, 2001).

We also studied the effects of rACC lesions on the mechanical allodynia produced in the SNI model of neuropathic pain. One week after SNI, mechanical thresholds were assessed with von Frey filaments, after which the animals underwent rACC lesion or sham surgery. After a 6 day recovery period, we retested mechanical thresholds of the ipsilateral and contralateral hindpaws. Again, we found no difference between the magnitude of mechanical allodynia in the lesion (*n = *9) and control (*n = *7) groups (Fig. 1E; RM 2-way ANOVA followed by Tukey’s multiple comparison test, *P* < 0.001). Further, lesions in the rACC did not alter the mechanical threshold of the uninjured paw (Supplementary Fig, 1; RM 2-way ANOVA followed by Tukey’s multiple comparison test, not significant).

Finally, Fig. 1F shows that rACC lesions (*n = *5) significantly reduced avoidance of the formalin-paired side of the CPA apparatus (CPA score = 16.26 ± 17.36 s, Student’s *t*-test, *P* < 0.001) compared to the control mice (CPA Score = −103.2 ± 12.70 s, *n = *7). Taken together, these findings demonstrate that rACC lesions in the mouse, as in the rat (Johansen *et al.*, 2001), significantly interfere with pain’s affective dimension, but do not reduce the sensory discriminative aspect of the pain experience.

### Biochemical consequences of peripheral nerve injury on GABAergic signalling in the rACC

**Figure 2:**
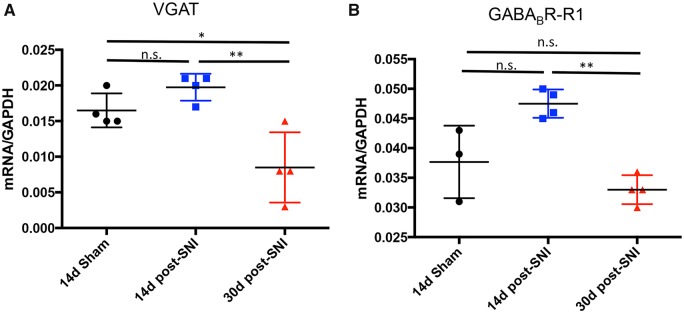
**Gene expression (qPCR) in the rACC after peripheral nerve injury.** (**A**) *Slc32a1*/VGAT mRNA decreases in nerve-injured mice (*n = *4), compared to sham (*n = *4), 30 days after SNI. All values were normalized to *Gapdh* (one-way ANOVA, Tukey’s multiple comparisons test; *P < 0.05; **P < 0.01). (**B**) *Gabbr1*/GABA-B-R1 mRNA is transiently upregulated in the rACC 14 days after SNI (**P < 0.01), but returns to baseline by 30 days post-injury (one-way ANOVA, Tukey’s multiple comparisons test; not significant).

To identify correlates of GABAergic dysfunction in the rACC in the setting of neuropathic pain, we used quantitative PCR (qPCR) to measure mRNA levels of several GABA signalling-associated genes. We compared sham (*n* = 4) and SNI-operated mice at 14 (*n = *4) and 30 days (*n = *4) after injury. We found no change of vesicular GABA transporter *Slc32a1* (VGAT) mRNA at 14 days post-SNI, but at 30 days post-SNI we recorded a 49% decrease (Fig. 2A; one way ANOVA, Tukey’s multiple comparisons test, *P = *0.0029). The decrease of *Slc32a1*/VGAT mRNA likely contributes to the reduction of GABA release in the ACC of nerve-injured rats (Narita *et al.*, 2011). Somewhat surprisingly, at 14 days post-SNI, we recorded a transient increase (26%) in *Gabbr1* mRNA, which codes for one of the GABA-B receptor subunits, but levels returned to baseline by Day 30 (Fig. 2B; One way ANOVA, Tukey’s multiple comparison test, *P = *0.0016). We detected no significant difference (one-way ANOVA, Tukey’s multiple comparisons test, *P* > 0.05, not significant) in mRNA levels for glutamate decarboxylase-65 (GAD65, now known as *Gad2*), glutamate decarboxylase-67 (GAD67, now known as *Gad1*), GABA transporter-3 (GAT3, now known as *Slc6a11*) or the GABA-A receptor alpha1 subunit (GABARα1, now known as *Gabra1*) (Supplementary Fig. 2).

### Transplanted MGE cells mature into GABAergic interneurons and integrate into host circuitry

**Figure 3:**
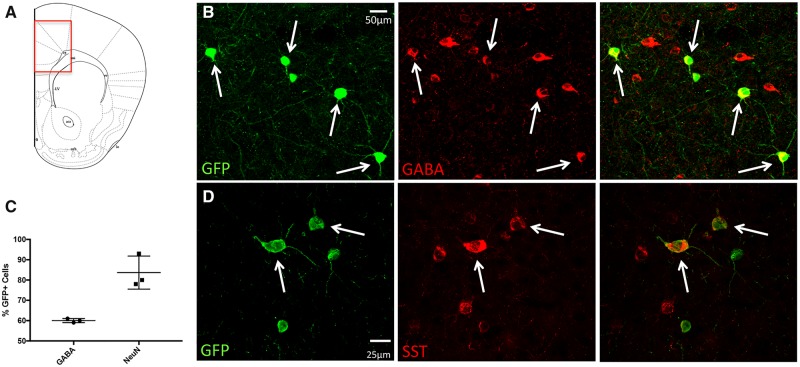
**MGE-cell transplants in the ACC of paclitaxel-treated mice.** (**A**) Schematic of MGE transplant location. (**B**) Thirty days after transplant, GFP+ (green) MGE cells express GABA (red). (**C**) The majority of transplanted cells are neuronal (NeuN+) and express GABA. (**D**) Some GFP+ MGE cells co-express somatostatin (SST; red), a marker of a subpopulation of cortical GABAergic interneurons.

Building upon the hypothesis that decreased inhibition in the ACC underlies pain aversiveness in different neuropathic pain conditions, here we asked whether increasing GABAergic tone, by transplanting GABAergic interneuron progenitor cells into the ACC, could reduce the ongoing pain/aversiveness. Although previous studies of the contribution of the ACC to pain aversiveness used peripheral nerve injury, or formalin-induced persistent pain models, in our experiments we used the paclitaxel-induced chemotherapy-model of neuropathic pain. We chose this model specifically because there is widespread hypersensitivity, which compared to the SNI model, increases the ability to demonstrate preference for a drug that reduces ongoing pain aversiveness. Furthermore, in the paclitaxel model of neuropathic pain there is also evidence that GABAergic signalling is disrupted in the ACC (Masocha, 2015; Nashawi *et al.*, 2016). We transplanted MGE cells bilaterally, 1 week after the paclitaxel treatment ended, at which time there is pronounced mechanical hypersensitivity of the hindpaws. Figure 3 shows a schematic of the injection site into the rACC (Fig. 3A) and examples of GFP+ MGE cells 30 days after transplant (Fig. 3B and D), at which time the cells migrated on average 850 μm in both the rostral and caudal directions. We estimate that only 2.0 ± 0.24% of the transplanted cells survived. This survival rate was likely underestimated due to the difficulty of counting cell clumps but is nevertheless consistent with our previous transplant studies (Braz *et al.*, 2012). Immunostaining of the GFP+cells with a pan neuronal marker (NeuN) demonstrated that ∼84% of the transplanted cells are neurons (Fig. 3C). We never found double labelling for astrocyte (GFAP) or microglial (Iba1) markers (data not shown). As Alvarez-Dolado *et al.* (2006) found no overlap with oligodendrocyte markers, we suggest that the remaining 16% were also neurons. This is especially true as not all neurons are NeuN positive (Mullen *et al.*, 1992). As expected, all transplanted cells expressed GAD67, manifest by GFP immunoreactivity. However, although only ∼60% immunostained for GABA, we presume that all are inhibitory (Fig. 3B and C). The incomplete GABA immunostaining, we suggest reflected antibody limitations. With respect to subtypes of cortical interneurons, we found that ∼31% of the transplanted cells were SST immunoreactive (Fig. 3D). Unexpectedly, we rarely record parvalbumin-immunoreactive transplanted cells (however, see below).

### MGE cells exhibit intrinsic properties of cortical inhibitory interneurons

**Figure 4:**
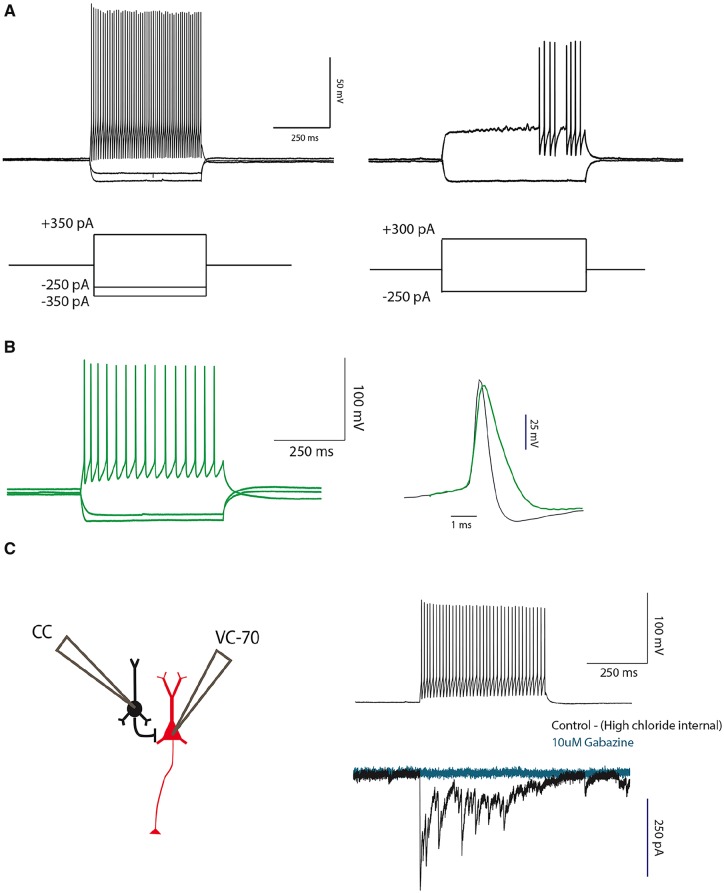
**Transplanted MGE cells are GABAergic and presynaptic to cortical pyramidal cells.** (**A**) Current-clamp recordings from GFP+ MGE neurons show fast spiking (*left trace*; *n = *8/18) and latent fast spiking (right trace; *n = *3/8). (**B**) Non-fast spiking MGE cells (*left trace*; *n = *10/18) MGE cells. *Right*: The difference in action potential shape between a fast (blue) and a non-fast spiking (green) neuron. (**C**) Schematic (*left*) shows the paradigm used to establish functional connectivity between MGE (black) and host (red) pyramidal neurons. Panel to the right shows action potential discharges evoked in the MGE neuron (*top*) and the resulting IPSC in a host pyramidal neuron (*bottom*, black), which was blocked by 10 µM gabazine (blue; *n = *3/17 of fast spiking neurons and 0/9 non-fast spiking neurons).

In these studies, we performed whole-cell patch clamp recordings from GFP+ cells in 400-μm thick coronal sections of the ACC, 30 days after transplant. In current-clamp mode, we identified all firing patterns characteristic of cortical inhibitory interneurons (Kawaguchi and Kondo, 2002). Most prominent are the fast-spiking (Rm < 350, interspike interval < 1.3) and latent-spiking phenotypes that are readily observed in parvalbumin (PV)-expressing cortical interneurons, (Fig. 4A; *n = *8). This finding suggests that despite the poor recovery of PV-immunoreactive transplanted neurons, some of the transplanted cells did, in fact, develop PV characteristics. As expected, we also recorded non-fast spiking GFP+cells (Fig. 4B; *n = *10), which likely correspond to the SST+population of interneurons (Kawaguchi and Kondo, 2002). See Table 1 in the Supplementary material for other electrophysiological parameters of the cortical MGE transplants.

We next performed random paired recordings from GFP+neurons and nearby host pyramidal neurons. We held the GFP+ and pyramidal neurons in current and voltage-clamp (−70 mV), respectively, the latter with a high chloride internal-pipette solution. Figure 4C, top right, shows that current injection into GFP+ neurons elicited a series of spikes in the interneurons and also induced a chloride current in 3/17 pyramidal neurons (Fig. 4C, bottom right), demonstrating that at least some of the transplanted MGE neurons were functionally connected to host pyramidal neurons. To determine whether GABA mediated these currents, we applied 10 μM gabazine (a GABA-A receptor antagonist) to the bath and observed blockade of the chloride current in the pyramidal neuron (Fig. 4C, right).

**Figure 5:**
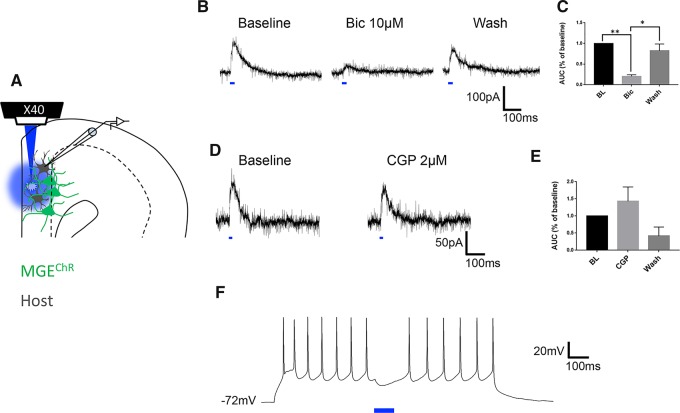
**MGE-evoked inhibition is GABA-A receptor mediated.** (**A**) Schematic illustrates the optogenetic approach to stimulate (blue light) populations of MGE cells (green) and study the pharmacology their inhibition of host pyramidal cells (black). (**B**) Optogenetic activation of MGE cells (blue bar) evokes bicuculline (Bic)-sensitive IPSCs in host pyramidal neurons: Baseline, after bicuculline and after a 10-min wash. (**C**) The magnitude of the evoked IPSCs (expressed as area under the curve) was significantly reduced by bicuculline and recovered to baseline (BL) after wash (*n = *10; baseline versus bicuculline, **P < 0.01; *P < 0.05 Friedman test with Dunn’s multiple comparison correction). (**D** and **E**) The GABA-B receptor antagonist CGP55845 did not alter the magnitude of the optogenetically-evoked IPSCs. (**F**) Optogenetic stimulation of MGE cells (blue bar; 100 ms), transiently blocked evoked firing in the host pyramidal neuron.

In a separate set of experiments, we studied acutely dissected brain slices (400 μm) from animals in which we had transplanted MGE neurons that express channelrhodopsin2 (ChR2) under the control of a forebrain specific enhancer (I12b) that targets GABAergic neurons. This arrangement made it possible to activate simultaneously a large population of transplanted MGE neurons (Fig. 5A). While holding pyramidal neurons in voltage-clamp (+10 mV), we applied a 20 ms light pulse (460 nm) and observed evoked inhibitory postsynaptic currents (IPSCs) in 12/19 host pyramidal neurons (IPSC amplitude: 106 ± 22 µA; mean ± SEM). In all neurons tested (*n = *10), bath application of the GABA-A receptor, bicuculline (10 µM) blocked the MGE-evoked IPSCs by 75 ± 3% (Fig. 5B and C baseline versus bicuculline, *P = *0.001; Freidman test with Dunn’s multiple comparison correction). Importantly, the amplitude of the optogenetically-evoked IPSCs returned to baseline after a 10-min wash, which removed the bicuculline (bicuculline versus wash, *P = *0.014; Freidman test with Dunn’s multiple comparison correction). The GABA-B receptor antagonist, CGP55845 (2.0 μM; *n = *7; Fig. 5D and E) had no effect. We conclude that MGE transplant-induced inhibition of the host neurons, as demonstrated previously in the adult mouse brain (Zipancic *et al.*, 2010; Casalia *et al.*, 2017) and spinal cord (Etlin *et al.*, 2016) is GABA-A receptor-dependent. Despite a report of GABA-B-mediated inhibition of some pyramidal cells by somatostatin-expressing interneurons (Urban-Ciecko *et al.*, 2015), the lack of effect of the GABA-B receptor antagonist is consistent with the limited expression of GABA-B receptors in cerebral cortex (Chu *et al.*, 1990),

Finally, we asked whether activation of MGE neurons inhibits current-evoked action potentials in host pyramidal neurons. We injected pyramidal neurons with sufficient current to induce action potential firing, and followed this with a 100-ms flash of blue light, to activate the ChR2. As illustrated in Fig. 5F, optogenetic activation of MGE neurons immediately inhibited action potential firing in the host pyramidal neurons, but only for the duration of the light exposure. Taken together, these data provide strong evidence that the transplanted MGE neurons successfully integrate into host ACC cortical circuitry, form GABAergic synapses and can block action potential firing in pyramidal neurons via the GABA-A receptor. Based on the time-locked inhibition that we observed, we propose that the inhibition involves a circuit-based synaptic inhibition, rather than pump-like diffusion of GABA from the transplanted cells.

### MGE cell transplants receive inputs from widespread brain regions

We used rabies virus tracing (Callaway and Luo, 2015) to identify presynaptic inputs to the transplanted neurons. In these studies we transplanted MGE cells that co-expressed the avian TVA receptor and rabies G protein. These MGE cells were harvested from triple transgenic mice (I12b-Cre × ROSA-tdTomato mice × lox-STOP-lox-TVA_RG mice). Thirty days later we injected the transplanted area with an avian-pseudotyped rabies virus that expresses GFP (Braz *et al.*, 2015*a*). This arrangement restricted infection of the rabies virus to the MGE cells. Importantly, because only the MGE neurons expressed the rabies glycoprotein, the rabies virus is trapped in the immediately presynaptic neuron, allowing identification of neurons that are monosynaptically connected, presynaptic partners of the MGE cells.

**Figure 6:**
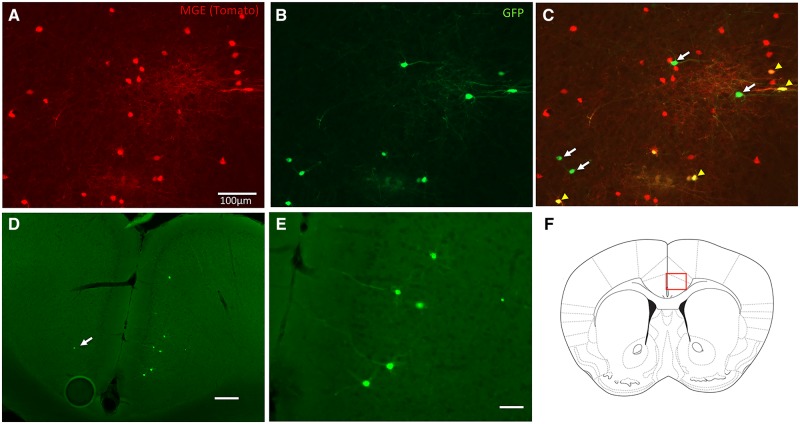
**Presynaptic inputs to transplanted MGE cells.** (**A**) Tomato- (red) and TVA-expressing MGE cells 30 days after unilateral transplant into the rACC. (**B** and **C**) Because the rabies-GFP-ΔG virus (green) is pseudotyped to express EnVa, this virus can only enter TVA-expressing starter cells (yellow cells; yellow arrowheads) and be retrogradely transported to immediately presynaptic neurons, which are only GFP-expressing (green, white arrows). (**D** and **E**) Retrogradely-labelled cells in the pACC (magnified in **E**) that are presynaptic to MGE cells transplanted in the rACC. Occasionally, we observed GFP+, presynaptic cells bilaterally (arrowhead in **D**), indicating that some MGE transplants receive bilateral inputs. (**F**) The schematic illustrates the location of retrogradely-labelled cells in the pACC. Scale bar = 75 μm except in **D** (200 μm).

Figure 6A shows an example of the MGE injection site. Some of the MGE cells only express tdTomato (red), indicating that they did not incorporate the rabies virus (green; Fig. 6B). In contrast, starter cells, which took up the rabies virus, are yellow (yellow arrowheads in Fig. 6C), as they co-express both tdTomato and GFP. Monosynaptically connected, presynaptic partners of the MGE neurons only express GFP (white arrows in Fig. 6C). Based on analysis of transneuronally labelled neurons in four mice, we observed consistent, albeit sparse, labelling of monosynaptic, presynaptic partners of the MGE cells, including GFP+ neurons in the ipsilateral, and less frequently contralateral, posterior ACC (pACC; Fig. 6D and E), the nucleus of the horizontal limb of the diagonal band, medial and intralaminar thalamic nuclei (primarily the mediodorsal nucleus), lateral hypothalamus and medial amygdala (Supplementary Fig. 3). These results compare well with descriptions from previous retrograde tracing studies that examined inputs to the cingulate cortex (Horikawa *et al.*, 1988) and demonstrate that there is axonal growth and connections with transplanted neurons from local as well as distant host neurons that normally target the ACC. Of course, the previous studies could not define the neuronal target of the projections to the ACC. Taken together with the electrophysiological analysis, we conclude that MGE cells successfully integrate into host circuitry, receive local and long distant neuronal inputs and exert a GABA-A mediated inhibitory control of host pyramidal neurons.

### MGE cell transplants into the rACC relieve ongoing pain, provided the cells do not also populate the pACC

**Figure 7:**
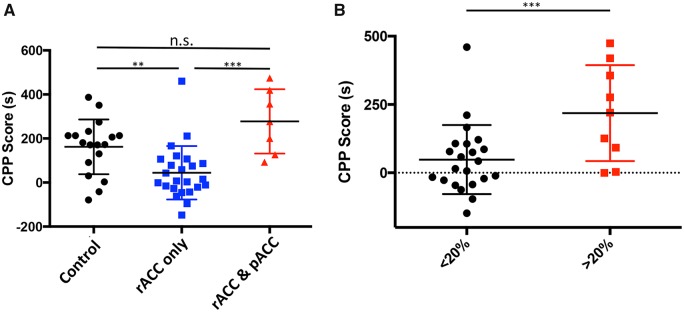
**Rostral and posterior ACC differentially influence pain aversiveness.** (**A**) MGE transplant-mediated selective inactivation of the rACC (rACC only; *n = *24) reduces preference for gabapentin (i.e. reduces pain aversiveness, **P < 0.001); preference is preserved after transplant-mediated inactivation of both rACC and pACC (*n = *7) or in control (DMEM-injected mice; *n = *18). (**B**) Mice in which > 20% of the transplanted MGE cells migrated into the pACC (*n = *9) exhibited a preference for gabapentin, whereas mice with < 20% of MGE cells (*n = *22) in the pACC showed no preference.

Here we examined the behavioural consequence of transplant-mediated recovery of inhibitory control in the ACC. Our studies specifically addressed the ability of MGE cell transplants to reduce the pain aversiveness (ongoing pain) characteristic of neuropathic pain. As noted above, systemic gabapentin is not inherently rewarding in uninjured animals. However, in the setting of ongoing pain, gabapentin does induce a preference in the CPP test as it is pain relieving and thus rewarding. It follows that if MGE transplantation is also pain relieving, i.e. reduces aversiveness, then there should not be a preference for gabapentin. Figure 7A shows that control animals (injected with DMEM only; *n = *18) showed an average CPP score of 162.1 ± 29.30 s, which indicates that gabapentin was, in fact, pain relieving. On the other hand, animals transplanted with MGE cells in the rACC had no preference for gabapentin (*n = *24; CPP score = 44.38 ± 24.78 s, *P = *0.0002). We conclude that the transplant, like gabapentin, induced pain relief. Importantly, at 30 days post-transplant, the MGE cells had no effect on paclitaxel-induced mechanical allodynia, which measures the sensory discriminative feature of the pain experience (Supplementary Fig. 4).

Analogizing between the cytoarchitectural regions of the human and the mouse ACC is difficult. In the present study, we focused on the rACC and pACC, which may correspond to rostral and posterior parts of area 24 in human cortex. Importantly, to facilitate localizing the distribution of transplanted cells, here we provided stereotaxic coordinates that can be readily replicated. As noted above, MGE cells often migrate considerable distances rostral and caudal to the injection site. As a result, we always recovered transplanted MGE cells (GFP+ neurons) in the rACC (stereotaxic coordinates: +2.46 to +1.18 mm from bregma), in the pACC (stereotaxic coordinates: +1.10 to +0.20 mm from bregma), some of which expressed somatostatin (Supplementary Fig. 5), in the secondary motor cortex (M2) and in the prelimbic cortex. Based on a *post hoc* analysis of the relationship between the distribution of transplanted cells and results in the CPP test, we unexpectedly detected a critical consequence of transplanted cells residing in the pACC. Specifically, in animals in which a large number of GFP+ transplanted cells migrated to the pACC (>150 cells), the preference for gabapentin persisted (*n = *7; CPP score = 92 ± 55.24 s), despite the fact that the rACC also contained significant numbers of transplanted cells. This result suggests that concurrent MGE-mediated inhibition of the pACC can counteract the analgesic effect of increasing inhibitory tone in the rACC (Fig. 7A; One way ANOVA Kruskal-Wallis test with Dunn’s multiple comparison correction; rACC only versus rACC and pACC, *P = *0.0002; control versus rACC and pACC, not significant). In fact, Fig. 7B illustrates that when more than 20% of the MGE cells migrated to the pACC, the animals exhibited preference for gabapentin. Importantly, this counteracting effect was limited to the pACC. Migration of cells into the prelimbic cortex and/or M2 regions did not influence the CPP score of MGE-transplanted mice (data not shown).

### Lesions of the pACC counteract the analgesic (anti-aversive) effect of rACC lesions

**Figure 8:**
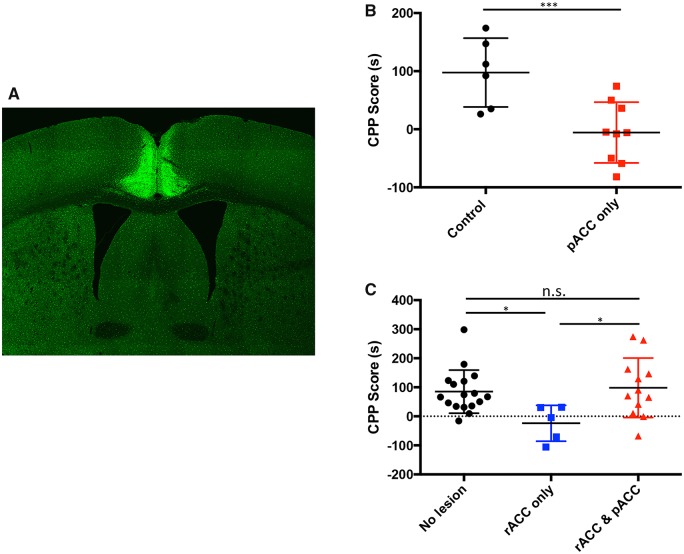
**Lesions restricted to the pACC relieve ongoing pain in paclitaxel-treated mice.** (**A**) Ibotenic acid lesion of the pACC increases Iba1 immunostaining (green), illustrating microglial activation. (**B**) Mice with ibotenic acid lesions limited to the pACC (*n = *9) do not show a preference for gabapentin compared to control animals (*n = *6). Data are presented as mean ± SEM, Student’s *t*-test: ***P < 0.001. (**C**) Ibotenic acid lesions of the rACC (*n = *5) also reduced preference for gabapentin (*P < 0.05), but the preference for gabapentin was preserved after combined rACC and pACC lesions (*n = *12) or in vehicle-treated control mice (*n = *17, one way ANOVA Kruskal-Wallis test with Dunn’s multiple comparison correction).

We next asked whether excitotoxic lesions of the rACC and pACC recapitulate the surprising results observed after MGE transplants that spanned the rACC and pACC. In separate groups of mice, we selectively ablated the rACC, the pACC, or made combined lesions of the rACC and pACC, 1 week after paclitaxel treatment. Seven days after the lesions (namely 14 days after initiating the paclitaxel), we monitored pain aversiveness using the gabapentin CPP paradigm. Control animals (*n = *17) that did not undergo a cortical lesion had a CPP score of 87 ± 17.99 s (Fig. 8C). Animals that had lesions limited to the rACC (*n = *5), as expected, did not exhibit a preference for gabapentin (CPP score=−23.97 ± 27.65 s, One way ANOVA Kruskal-Wallis test with Dunn’s multiple comparison correction, *P = *0.0137). And consistent with our findings after MGE transplants that included the pACC, we found that mice with combined lesions of the rACC and pACC (*n = *12) did show a preference for gabapentin, with CPP scores of 98.33 ± 29.58 s. These scores did not differ from what we recorded in control (medium-injected) mice, namely those without lesions (*n = *6; CPP Score = 97.67 ± 24.19 s). Surprisingly, however, mice that had lesions limited to the pACC (*n = *9) did not show a preference for gabapentin (Fig. 8B, CPP score = −5.55 ± 17.4 s, Student’s *t*-test, *P = *0.0002). Because rats find acute formalin aversive even after undergoing pACC lesions (Johansen *et al.*, 2001), this result was not expected.

To assess whether the pACC might contribute to pain aversiveness in a more prolonged inflammatory pain setting, we examined the effect of a lidocaine ring block (2.0% in 50 µl) around a complete Freund’s adjuvant (CFA; 50%)-induced inflamed hindpaw. We could not use non-steroidal anti-inflammatory drugs because their long duration of action would compromise testing in the CPP apparatus. One day after a hindpaw injection of CFA, the mice were conditioned for 2 days with lidocaine and on the third day (peak day of CFA-induced inflammatory pain) they were tested for preference. Not only did the mice show a small, but significant preference for the lidocaine paired chamber, but prior lesion of the pACC or of the rACC also prevented preference development [CPP scores (in seconds) Sham: 30.6 ± 10.5; rACC lesion: 3.1 ± 13.0; pACC lesion:−13.8 ± 13.4; *P = *0.048; one-way ANOVA]. These results indicate that the pACC, at least in the mouse, contributes to aversiveness in both neuropathic and some inflammatory pain settings. We conclude that inactivation of the rACC or pACC, either by MGE-mediated inhibition or by excitotoxic ablation, is pain relieving, but that the pain relief can be reversed by concurrent inactivation of the rACC and pACC.

## Discussion

As loss of GABAergic inhibition within the rACC is a major contributor to ongoing neuropathic pain, particularly to its aversive features, increasing GABAergic tone in the rACC is a logical approach to neuropathic pain management. Systemic or even local administration of an analgesic that targets the rACC may be therapeutic, but not ideal, not only because of adverse side effects, but also because the analgesic must be chronically administered directly into cortical tissue. To this end, Bannister *et al.* (2017) demonstrated recently that injection of gabapentin into the rACC is, in fact, pain relieving, but long-term pharmacological relief at the level of the cortex would be difficult. An ideal pharmacotherapy would target the dysfunctional circuit, rather than flooding the cortex. In this regard, a cell transplant-based approach more closely recapitulates cortical inhibitory controls. Here we demonstrate that transplants of GABAergic progenitor cells from the MGE enhance inhibitory tone in the ACC and selectively regulate pain aversiveness. The transplants integrate into host circuitry and form functional inhibitory synapses onto pyramidal neurons, the major ACC output neuron. Note that we do not believe that the transplant utility involved opening of a plasticity window that permitted injury-induced GABAergic inhibitory regulation in the adult (Tang *et al.*, 2014). Rather we suggest that circuit inhibition is required, as evidenced by the failure of spinal cord transplants taken from VGAT mutant mice, which have no inhibitory potential, to relieve chemotherapy-induced hypersensitivity (Braz *et al.*, 2015*b*).

Viral tracing demonstrated that a variety of host neurons, from thalamus, hypothalamus and amygdala target the transplanted cells. Of particular importance, and comparable to the effects of rACC lesions in the rat, we found that MGE cell-mediated inhibition of the rACC significantly reduced pain aversiveness in the CPP test, without affecting the associated mechanical allodynia. Unexpectedly, although selective lesions of the rACC or pACC were pain relieving, combined inhibitory control of the rACC and pACC, either by MGE transplant or chemical ablation, was not. Although previous studies emphasized the differential contribution of the mid and posterior cingulate cortex (Vogt, 2005, 2016; Tan *et al.*, 2017), our new findings are consistent with previous studies reporting that subregions within the ACC itself, namely the rACC and pACC, must be taken into consideration when examining circuits that influence pain aversiveness in the setting of chronic neuropathic pain (see below).

### MGE-mediated inhibition of ACC pyramidal neurons

Using patch electrophysiology we found that many MGE cells displayed a non-fast spiking firing pattern, characteristic of the somatostatin subset of GABAergic inhibitory interneurons (Kawaguchi and Kondo, 2002; Alvarez-Dolado *et al.*, 2006). In fact, 31% of the cells immunostained for somatostatin. Although we rarely detected parvalbumin-immunoreactive MGE neurons, we did record from some MGE cells with intrinsic firing patterns typical of PV-positive GABAergic interneurons (Kawaguchi and Kondo, 2002), including both fast-spiking and latent fast-spiking neurons. Regardless of the subtype of transplanted neurons, it is clear that the functional consequence of transplantation, namely pain relief, involves a profound GABA-A, but not GABA-B receptor-mediated inhibition of pyramidal neurons. These results are consistent with behavioural studies demonstrating that injection of GABA-A receptor, but not GABA-B receptor agonists into the rACC of nerve-injured mice reduces the aversiveness produced by painful stimuli (LaGraize and Fuchs, 2007).

Importantly, MGE transplants provide a significant therapeutic advantage over ablative strategies that inevitably destroy axons of passage that course through the ACC. Moreover, there is no opportunity for modulation of the ACC output after its surgical ablation. The same is true for microinjection of GABA, which mimics volume transmission. In contrast, as diverse brain regions integrate synaptically with the transplanted cells, there likely is significant opportunity for regulated release of GABA from the transplanted neurons and thus a more subtle response to pain-provoking conditions. In this respect, MGE transplants can reduce nerve injury-induced hyperactivity of ACC circuits, without taking the ACC completely offline.

### Rostral versus posterior ACC

Previous studies demonstrated significant differences in the contribution of the rACC and pACC to pain processing. For example, in the rat, using a conditioned place aversion paradigm Johansen *et al.* (2001) demonstrated that excitotoxic lesions of the rACC, but not the caudal ACC, i.e. the pACC, abolished the aversiveness produced by an intraplantar injection of formalin. The authors concluded that the rACC, but not the pACC, contributes to the affective component of the pain percept. Importantly, however, those experiments only assessed the contribution of the pACC to the aversiveness provoked by an acute noxious stimulus, namely intraplantar formalin. Our findings indicate that the pACC contribution differs in the setting of ongoing pain. Conceivably, as pain persists a chronification process recruits additional brain regions to the processing of pain aversiveness. Alternatively, as the posterior part of the ACC becomes hyperactive following nerve injury, in both humans (Baliki *et al.*, 2011) and rodent models (Wei and Zhuo, 2001; Xu *et al.*, 2008; Kang *et al.*, 2012; Chen *et al.*, 2014), our findings suggest that there are differences in pain aversiveness associated with inflammatory (namely, formalin-induced) versus neuropathic pain. On the other hand, there are clearly differences in the contribution of the pACC to prolonged inflammatory pain, namely that produced by CFA. The fact that mice with pACC lesions did not exhibit a preference for a local anaesthetic ring block of the inflamed paw indicates that the pACC, at least in the mouse, contributes to aversiveness in both neuropathic and some inflammatory pain settings.

We have no simple explanation for our observation that concomitant inhibition of the rACC and pACC, by MGE transplants or combined surgical ablation, did not eliminate the preference for gabapentin. Clearly, other regions that underlie pain aversiveness must come into play when both the rACC and pACC are disrupted. Likely candidates are the amygdala and insular cortex as: (i) both regions receive direct input from the nociresponsive mediodorsal thalamus (Krettek and Price, 1977); (ii) both regions normally process aversiveness in other settings, i.e. fear, hunger, thirst (LaBar *et al.*, 1998; Tataranni *et al.*, 1999; Del Parigi *et al.*, 2002; Egan *et al.*, 2003), and even the dysphoria provoked by a kappa opioid receptor agonist, which is not disrupted by rACC lesions (Johansen *et al.*, 2001); and (iii) both regions are reciprocally connected with the rACC and pACC (Fisk and Wyss, 1999). Further studies are clearly needed to determine how the rACC-pACC circuits contribute to the balance of network activity arising from disparate brain regions that normally process aversiveness unrelated to persistent pain.

Although direct injection of gabapentin into the rACC is pain relieving (Bannister *et al.*, 2017), the fact that systemic gabapentin-induced analgesia is preserved after combined ablation of both the rACC and pACC shows that its analgesic action does not require interaction with rACC or pACC circuits. One possibility, of course, is that systemic gabapentin targets brain regions (e.g. insula or amygdala) that generate the newly established aversiveness evoked by rACC-pACC inhibition. It is more likely, however, that gabapentin is effective in this condition because it regulates nociceptive input at a lower level of the neuroaxis. In fact, we recently demonstrated that the analgesic action of supraspinal gabapentin involves activation of brainstem noradrenergic controls that inhibit the processing of nociceptive messages at the spinal cord level (Juarez-Salinas *et al.*, 2018).

## Conclusion

Here we demonstrate that MGE cell transplants increase GABAergic inhibitory control in the rACC and reduce ongoing pain aversiveness. Unexpectedly, we found that pain aversiveness persists when there is concurrent inhibition (or ablation) of the rACC and pACC. We propose a novel contribution of the pACC to pain aversiveness in the setting of persistent (neuropathic) pain. Taken together our findings support the introduction of cell transplants for the long-term management of ongoing pain aversiveness. Clearly a major advantage of the transplant approach over ablation is that the latter approach is not only invasive, but also irreversible. Lastly, to the extent that the organization of murine ACC circuitry mimics that in the human, our results also underscore the importance of targeting particular ACC subregions in future pain management approaches.

## Funding

This work was supported by NIH Grant: NS097306 (A.I.B.), Wellcome Trust and MH100292 (V.S.).

## Competing interests

A.I.B. serves on the Scientific Advisory Board of Neurona Therapeutics.

## Supplementary Material

awz203_Supplementary_Data

## Abbreviations

CPA: conditioned place avoidance
CPP: conditioned place preference
MGE: medial ganglionic eminence
p/rACC: posterior/rostral anterior cingulate cortex
SNI: spared nerve injury
