# Supplementary material for: GABAergic cell transplants in the anterior cingulate cortex reduce neuropathic pain aversiveness
Source: Brain. 2019 Jul 18;142(9):2655–69. doi: 10.1093/brain/awz203 (PMC6752168; doi:10.1093/brain/awz203)
Supplement: awz203_Supplementary_Data [file awz203_supplementary_data.pdf]

SUPPLEMENTARY MATERIAL

SUPPLEMENTARY METHODS:

Study design:

Prior to treatment mice housed at 5/cage were randomly assigned to either control or MGE transplant protocol. After the procedure (medium or MGE cells), the mice were returned to a cage that contained mice from both groups (5 total per cage). The same protocol was used to randomize assignment of mice to either control or ablation experiment. The experimenter who performed the behavioral study was blind to treatment group. Before determining CPP scores, targeting of the ACC transplant or lesion was assessed. Twenty-one mice with incomplete lesions were not included in the final analysis and 3 mice were excluded from the MGE-gabapentin CPP study because the controls did not show a preference (CPP) for gabapentin. If any number of transplanted cells were recorded in the correct target, whether or not some cells were clustered, these mice were included in the final analysis.

Number of animals:

Experiment	Sample size	# groups	# samples	# mice
**Behavior**				
Baseline Mechanical	7-9 mice	2	16	16
Baseline Thermal (Heat)	6-13 mice	2	19	19
Formalin (ACC ablation)	9-12 mice	2	21	21
Formalin (ACC ablation plus CPA)	5-7 mice	2	14	14
CFA (ACC ablation plus CPP for lidocaine)	6-8 mice	3	21	21
CPP for gabapentin (ACC ablation)	5-17 mice	5	49	49
ACC transplants (CPP for gabapentin)	9-22 mice	3	49	49
**Electrophysiology**				
ACC transplants: Paired neuron recordings	17-18 cells	3	53	4
ACC transplants: Optogenetic stimulation	7-19 cells	2	29	12
**Other**				
Immunohistochemistry	3 mice	1	3	3
Rabies tracing	4 mice	1	4	4
qPCR	4 mice	3	12	12
**Total**				224

Electrophysiology: slice preparation and recordings

The composition of the dissection solution was (in mM): NMDG 93, KCl 2.5, NaH_2PO_4 1.2, $NaHCO_3$ 30, HEPES 20, Glucose 25, sodium ascorbate 5, Thiourea 2, sodium pyruvate 3, $MgSO_4$-$7H_2O$ 10, $CaCl_2$-$2H_2O$ 0.5. The composition of the recovery solution was (in mM): NaCl 92, KCl 2.5, NaH_2PO_4 1.2, $NaHCO_3$ 30, HEPES 20, Glucose 25, sodium ascorbate 5, thiourea 2, sodium pyruvate 3, $MgSO_4$-$7H_2O$ 2, $CaCl_2$-$2H_2O$ 2 (Etlin et al., 2016; Tanaka et al., 2008). On the day of the experiment, 8-9 week-old mice, 2-3 weeks following transplantation of embryonic MGE cells, were killed with an overdose of Avertin (Sigma-Aldrich, USA). Following transcardial perfusion with 8 ml of dissection solution the brain was quickly removed, mounted in a Vibratome (VT1200, Leica, USA) bath filled with ice-cold dissection solution and 4 transverse or parasagittal sections were cut. The sections were transferred to a glass beaker containing heated (37°C) recording or HEPES-based recovery solution. The sections recovered in heated solution for at least 1 hour before use. Next, the sections were transferred to a recording chamber (Automate Scientific, CA, USA) under an upright fluorescent microscope (Nikon E600FN, Japan) and superfused with recording solution at a rate of 1.0 ml/min. Sections were viewed with a CCD digital camera (Hamamatsu Inc., Japan or DAGE-MTI Inc., USA).

Patch electrodes were pulled on a horizontal pipette-puller (Sutter Instrument, USA) from thin-wall, fire-polished, borosilicate glass filaments to yield an impedance of 6-8MΩ. Patch electrodes (tip resistance = 2–6 MOhms) were filled with (in mM): 130 K-gluconate, 10 KCl, 10 Hepes, 10 EGTA, 2 MgCl, 2 MgATP, and 0.3 NaGTP (pH adjusted to 7.3 with KOH). All recordings were at 32.5 ± 1 °C. Series resistance was usually 10–20MΩ, and experiments were discontinued above30 MΩ. In some experiments, the composition of the pipette solution differed as follows: (in mM): K-methane sulfonate 140, NaCl 10, $CaCl_2$ 1.0, EGTA 1.0, HEPES 10, Mg-ATP 5.0, NaGTP 0.5.

Cells were visualized under near-IR differential interference contrast illumination and approached with a micromanipulator (Sutter Instrument, CA, USA) while monitoring the resistance in voltage-clamp mode using the "Membrane Test" module of pClamp10 software (Molecular Devices, CA, USA). To prevent clogging of the tip, we applied positive pressure to the pipette via a 1.0ml syringe. After a seal was established with a cell, we ruptured its membrane by gently applying negative pressure to the pipette to establish a whole-cell configuration. We did not correct for leak or junction potentials. Current and voltage signals were amplified using a DC amplifier (MultiClamp 700) and digitized using Digidata 1440a system (Molecular Devices, CA, USA) at 10kHz and stored for subsequent off-line analysis.

For recordings of inhibitory postsynaptic currents (IPSCs) in pyramidal neurons, a high -chloride internal solution was used that contained (in mM): 120 CsCl, 15 $CsMeSO_4$, 8 NaCl, 0.5 EGTA, 10

HEPES, 2 MgATP, 0.5 Na_3GTP. All recordings were made at 32–34°C. Series resistance was compensated in all current clamp experiments and monitored throughout recordings. Recordings were discarded if Rs changed by >25%.

Drug application

All drugs were purchased from Sigma Aldrich (USA) and stored as stock solution at -20°C. The drugs were applied through a perfusion system equipped with TTL-driven pinch-valves (Automate Scientific, CA, USA). The TTL signal used to open the valve and start the drug infusion was delivered via the pClamp10 software and also fed as an analog input to the digitizer, which ensured synchronization of the injection with the recordings.

Optogenetic stimulation

In some experiments we used a TTL-driven white LED (Sutter Instrument, USA) to excite the neurons or terminals expressing the light sensitive protein Channelrhodopsin 2. The white light was filtered to generate a 460nm wavelength. The light pulse was delivered through the X40 objective of the microscope with pulse duration of 5-20ms at 0.1Hz. The TTL signal that activated the light pulse was incorporated into the acquisition protocol and also fed as an analog input to the digitizer.

qPCR

We used the following primers to quantify the mRNA levels for GAD65 (Forward: CAG CAG TGC CCA GGC TCA TCG; Reverse: GGT GGT TCC AGC TGT GGC ACT C), GAD67 (Forward: CCG CCA CAA ACT CAG CGG CA; Reverse: TGG CGG CCA CAC TGA ATC GC), GABA$_A$R-□1 (Forward: TGG CCC ACA ACA TGA CCA TGC C; Reverse: ACG GCG TGG CTC TCT GGT CC), GABA$_B$R-R1(Forward: ACC CTG CCA ACA CCC GAA GC; Reverse: CGC ACT CCT GAA CGG CCA CC), GAT3 (Forward: GCT TGG GGC TAC GGC ATC GG; Reverse: GCC GTC GCC CTT GAC CTT GG), VGAT (Forward: AGA TCA CGG CGT GGG AAG CG; Reverse: CCT TCC ACA ATG CCA AAG TT) and GAPDH (Forward: GGG TGT GAA CCA CGA GAA AT; Reverse: CCT TCC ACA ATG CCA AAG TT)

Conditioned place preference

We used a 3-chambered custom designed apparatus (Tap Plastics). Each chamber had different visual (dots vs. stripes), olfactory (lemon vs. vanilla extract) and flooring (smooth vs. rough) cues, which distinguished the two end chambers. The neutral middle chamber had no cues that distinguished it. During habituation sessions, and on Pretest or Test days, the mice were always placed first into the neutral chamber.

On Day 1 in the afternoon (3:30PM – 4:00PM), the mice were habituated with full access to all chambers apparatus. On Day 2, in the morning the mice were habituated to the environment (9:00AM – 9:30AM), and in the afternoon their baseline preference for each chamber was recorded, for 30 min ("Pretest"). Importantly, in establishing whether there was a preference for each chamber, only the second 15 min of the video was scored. After Pretest readings were determined, a presumptive "pain reliever" (gabapentin or lidocaine) was paired with the less preferred chamber, and saline was paired with the more preferred chamber. On the conditioning days, Day 3 and 4 (morning; 9:00AM), saline (volume equivalent to that required for the test drug injection) was administered and the mouse restricted for 30 min to one of the two chambers. In the afternoon (3:30PM) the mice were injected with the "pain reliever" and restricted (immediately after injection of lidocaine or 45 minutes after injection of gabapentin) to the other chamber for 30 min. On Day 5, the "Test" day, the mice were placed into the middle chamber and allowed to roam freely between the 3 chambers of the apparatus, after which their preference for each chamber during the second 15 min of the trial was recorded. To calculate the CPP Score, we subtracted the time (seconds) spent in each chamber of the box on the Pretest day from that of the Test day (CPP Score = Test-Pretest).

SUPPLEMENTARY MATERIAL

Table 1: Electrophysiological parameters of MGE transplants in the rACC. RMP: resting membrane potential; Rm: resistance; AP: action potential; AHP: after hyperpolarization; ISI: interspike interval; SD: standard deviation

	Mean	SD	N	SEM
RMP (mV)	-70.0	3.22	18	0.76
Rm (Mohm)	466.5	354.48	18	83.55
Time constant (ms)	6.52	1.47	18	0.36
AP threshold (mV)	-35.77	4.71	18	1.12
AP amplitude (mV)	25.51	6.78	18	1.59
AHP amplitude (mV)	20.61	6.20	18	1.46
ISI (ms)	10.22	3.67	18	0.95

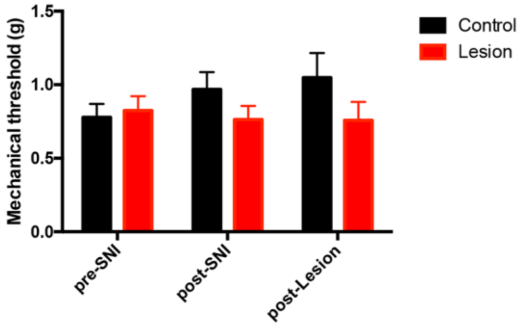

Supplementary Figure S1: Neither rACC lesion (N = 9, red bars) nor a saline control injection into the rACC (N = 7, black bars) altered baseline mechanical thresholds of the uninjured hindpaw (RM 2-way ANOVA, Tukey's multiple comparisons test).

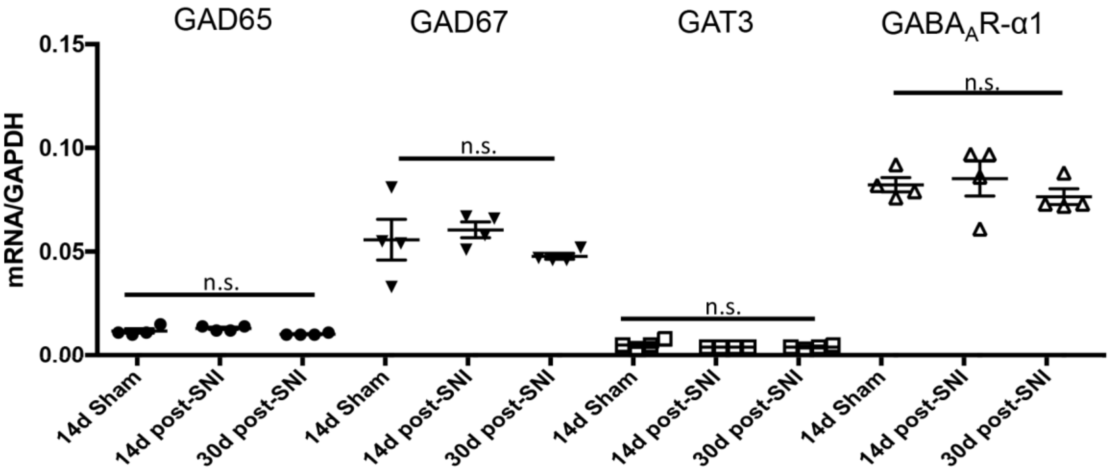

Supplementary Figure S2: rACC mRNA levels for GAD65, GAD67, GAT3 and GABA-A-α1 did not change after nerve injury (One-way ANOVA, Tukey's multiple comparisons test, n.s.). Data are represented as mean ± s.e.m.

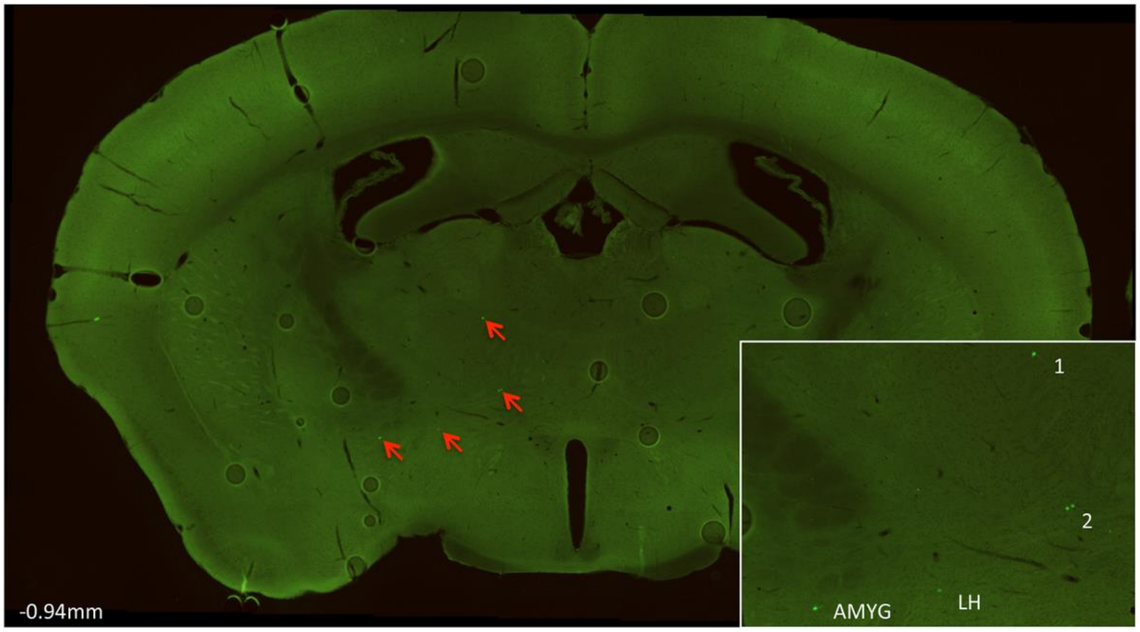

Supplementary Figure S3: Coronal brain section (-0.94mm from Bregma) illustrates retrogradely labeled GFP+ (green) neurons in the thalamus (1,2), lateral hypothalamus (LH) and amygdala (AMYG), all of which are presynaptic to MGE cells transplanted in the rACC. Similar patterns of labeling were seen across 3 different mice. Scale bar = 500µm

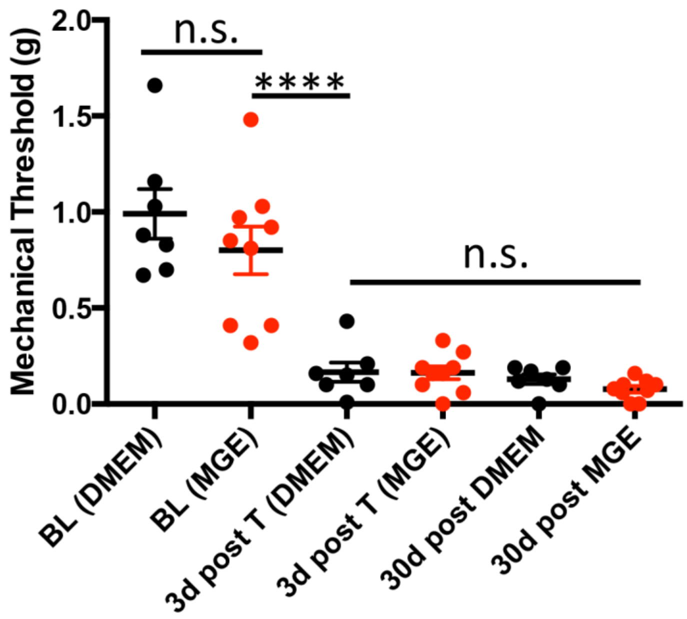

Supplementary Figure S4: MGE transplants in the ACC do not alter mechanical allodynia in paclitaxel-treated mice

Mechanical withdrawal thresholds of control (DMEM-injected) and MGE-transplanted mice at baseline (BL), 3 days after paclitaxel (T) and 30 days post transplant (MGE). All mice received paclitaxel, and 1 week after the last injection received either DMEM (control) or MGE-cell transplants into the rACC. Paclitaxel-induced mechanical allodynia was preserved in control and transplanted mice at the 3 and 30 day time point. Neither group showed a significant difference in their 3d post-T or 30d post treatment (DMEM or MGE) mechanical thresholds (RM 2-way ANOVA , Tukey's multiple comparisons test, ; ****p =< 0.0001; n.s. not significant). Data are represented as mean ± s.e.m.

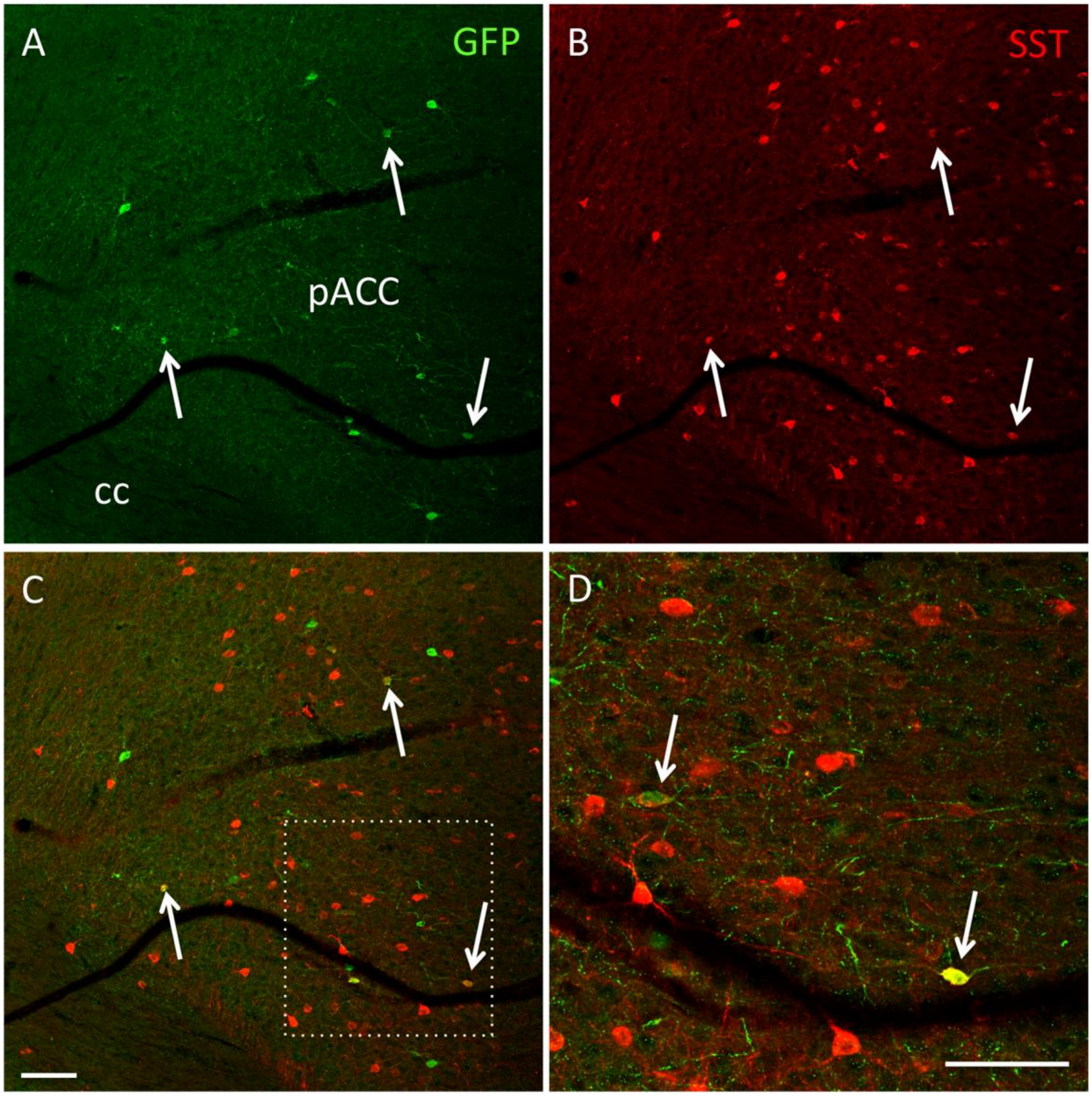

Supplementary Figure S5: Some GFP+ transplanted MGE cells (green in A,C,D) that migrated into the pACC differentiated into somatostatin-expressing interneurons (SST; red in B-D). Arrows point to GFP+/SST+ double-labeled MGE cells. (D) High magnification of the white square in (C). cc: corpus callosum. Scale bar equals 100 μm.
